# Involvement of 5′AMP-Activated Protein Kinase (AMPK) in the Effects of Resveratrol on Liver Steatosis

**DOI:** 10.3390/ijms19113473

**Published:** 2018-11-05

**Authors:** Jenifer Trepiana, Iñaki Milton-Laskibar, Saioa Gómez-Zorita, Itziar Eseberri, Marcela González, Alfredo Fernández-Quintela, María P. Portillo

**Affiliations:** 1 Department of Nutrition and Food Sciences, University of the Basque Country (UPV/EHU), 01006 Vitoria, Spain; jenifer.trepiana@ehu.eus (J.T.); inaki.milton@ehu.eus (I.M.-L.); saio86@hotmail.com (S.G.-Z.); itziar.eseberri@ehu.eus (I.E.); mariapuy.portillo@ehu.eus (M.P.P.); 2 Nutrition and Obesity Group, Lucio Lascaray Research Institute, 01006 Vitoria, Spain; 3 Biomedical Research Networking Centres, Physiopathology of Obesity and Nutrition (CIBERobn), Institute of Health Carlos III, 28029 Madrid, Spain; 4 Nutrition and Food Science Department, Faculty of Biochemistry and Biological Sciences, National University of Litoral and National Scientific and Technical Research Council (CONICET), 3000 Santa Fe, Argentina; maidagon@fbcb.unl.edu.ar

**Keywords:** resveratrol, AMPK, hepatocyte, liver, steatosis

## Abstract

This review focuses on the role of 5′AMP-activated protein kinase (AMPK) in the effects of resveratrol (RSV) and some RSV derivatives on hepatic steatosis. In vitro studies, performed in different hepatic cell models, have demonstrated that RSV is effective in preventing liver TG accumulation by activating AMPK, due to its phosphorylation. These preventive effects have been confirmed in studies conducted in animal models, such as mice and rats, by administering the phenolic compound at the same time as the diet which induces TG accumulation in liver. The literature also includes studies focused on other type of models, such as animals showing alcohol-induced steatosis or even steatosis induced by administering chemical products. In addition to the preventive effects of RSV on hepatic steatosis, other studies have demonstrated that it can alleviate previously developed liver steatosis, thus its role as a therapeutic tool has been proposed. The implication of AMPK in the delipidating effects of RSV in in vivo models has also been demonstrated.

## 1. Introduction

The most benign form of non-alcoholic fatty liver disease (NAFLD) is known as simple hepatic steatosis, and it consists of excessive fat accumulation in the liver. This hepatic alteration is considered to be the main contributor to chronic liver disease development in western societies. Moreover, the prevalence of NAFLD is expected to increase with the greater incidence of metabolic syndrome and obesity, which are closely related to hepatic steatosis [1]. Indeed, hepatic steatosis has been proposed as the hepatic manifestation of metabolic syndrome. This health alteration is defined as an intrahepatic triglyceride (TG) accumulation greater than 5% of the liver weight, or by ≥5% of hepatocytes showing TG content [2].

5′AMP-activated protein kinase (AMPK) is a master regulator of energy homeostasis, activated by low cellular energy status [3,4]. It restores energy balance during metabolic stress both at cellular and physiological levels [5]. Under conditions of energy depletion, the activation of AMPK leads to the inhibition of ATP-consuming pathways (e.g., fatty acid synthesis, cholesterol synthesis, and gluconeogenesis), and the stimulation of ATP generating processes (e.g., fatty acid oxidation and glycolysis), thus restoring overall cellular energy homeostasis [6,7,8]. The activation of AMPK is regulated by its heterotrimeric structure, which consists of a catalytic subunit α (α1 and α2), a regulatory and structurally crucial β subunit (β1 and β2), and a regulatory subunit γ (γ1, γ2, and γ3), with unique tissue and species-specific expression profiles [3]. Given its key role in regulating energy balance, AMPK may have therapeutic interest for the treatment of diseases in humans, such as insulin resistance, type 2 diabetes, obesity, NAFLD, cardiovascular diseases, and cancer [3,4,9].

**Figure 1:**
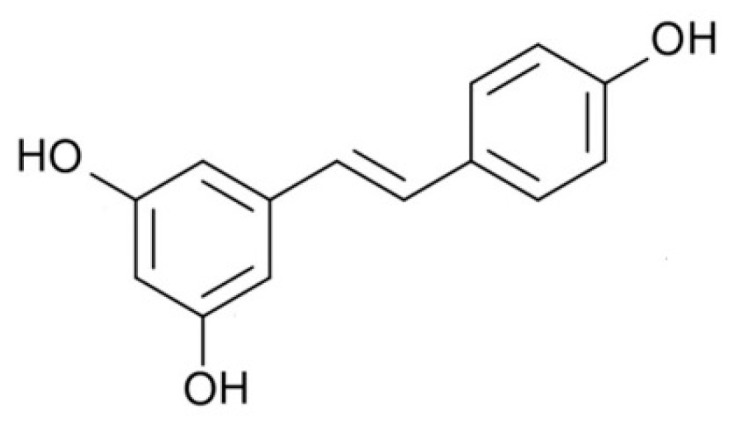
Chemical structure of *trans*-resveratrol (3,5,4′-trihydroxy-*trans*-stilbene).

Several molecules have been identified as AMPK activators. Among these molecules, resveratrol (RSV; 3,5,4′-trihydroxy-*trans*-stilbene) (Figure 1), a non-flavonoid polyphenol, has generated great interest [10]. Numerous studies have been carried out using RSV and different models of obesity and liver steatosis in rodents [2,11]. The vast majority of preclinical studies have demonstrated that RSV is able to prevent liver TG accumulation, although more evidence is still needed with regard to human beings [12,13,14].

**Figure 2:**
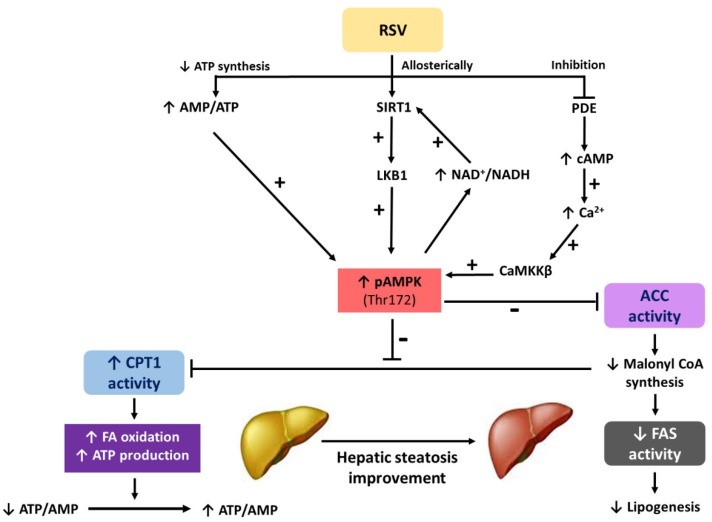
Effects of resveratrol (RSV) on hepatic steatosis improvement through 5′AMP-activated protein kinase (AMPK) activation. ACC: acetyl-CoA-carboxylase, AMPK: 5′AMP-activated protein kinase, AMP: adenosine monophosphate, ATP: adenosine triphosphate, cAMP: cyclic adenosine monophosphate, CaMKKβ: Ca^2+^/calmodulin-dependent protein kinase kinase β, CPT1: carnitine palmitoyltransferase 1, FA: fatty acid, FAS: fatty acid synthase, LKB1: liver kinase B1, NAD^+^: oxidized nicotinamide adenine dinucleotide, NADH: reduced nicotinamide adenine dinucleotide, PDE: phosphodiesterases, RSV: resveratrol, SIRT1: silent information regulator 1. (+: activation, −: inhibition, 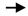: regulation direction; 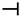: regulation inhibition).

As far as RSV-mediated AMPK activation is concerned, the role that is played in this process by sirtuin 1 (SIRT1) must be highlighted. Indeed, in both cases (AMPK and SIRT1), their regulation occurs in response to similar stimuli, namely nutrient availability and energy expenditure [15,16]. In this regard, while SIRT1 activation is enhanced by changes in the NAD^+^/NADH ratio (Figure 2), the activation of AMPK is mediated by changes in the AMP/ATP ratio [16]. In fact, although it is widely accepted that both molecules regulate the activation of each other [17], no consensus has been reached regarding which is activated first. In this regard, while some authors have reported that SIRT1 activates AMPK through the deacetylation of liver kinase B1 (LKB1, Figure 2) [18,19], other authors have suggested that AMPK activation results in an increased NAD^+^/NADH ratio, and thus, in greater SIRT1 activation [16,20]. In a study that was carried out in brains from Alzheimer’s disease patients, the authors observed that RSV increased cytosolic Ca^2+^ levels and so increased AMPK activation via calcium/calmodulin-dependent protein kinase kinase-β (CaMKKβ, Figure 2) [21]. In line with this study, Park et al. analyzed the metabolic diseases that are associated with aging, reporting that RSV increased cyclic adenosine monophosphate (cAMP) through phosphodiesterase (PDE) inhibition, without activating adenylyl cyclase activity directly. In fact, PDE inhibition prevented diet-induced obesity by the CaMKKβ/AMPK/SIRT1 pathway (Figure 2) [22].

This review focuses on the role of AMPK in the effects of RSV and some RSV derivatives on hepatic steatosis (Figure 2). For this purpose, in vitro and in vivo studies have been included.

## 2. In Vitro Studies

A wide range of in vitro studies have been performed to date using different hepatic cell models, such as human hepatoma cells (HepG2), murine hepatocytes (Hepa 1–6), rat hepatoma cells (H4IIEC3) or even primary hepatocytes. In some cases, hepatocyte TG accumulation was induced by including large amounts of glucose, insulin, free fatty acid (FFA) and/or alcohol in the incubation medium. These simulate the induction of fatty liver by following an obesogenic dietary pattern, as in the case of studies addressed to animal models, and imitate the situation that very often takes place in humans from western societies. In addition other models, such as hepatocytes incubated with fatty acids in the incubation medium and hepatocytes treated with an agonist of the transcription factor liver X receptor (LXR), have also been used (Table 1).

Zang et al. [23] examined the effect of several polyphenols, including RSV, on AMPK activity and lipid levels in human hepatoma HepG2 cells. In a previous in vitro study, these authors had reported that exposing HepG2 to elevated glucose (30 mmol/L) for 24 h induced decreased AMPK and acetyl CoA carboxylase (ACC) phosphorylation, as well as hepatocellular lipid accumulation [30]. Then, in the following study [23], when HepG2 were incubated with RSV (10 µmol/L) for 24 h, this treatment significantly stimulated ACC phosphorylation and the activity of AMPKα1, which is the predominant isoform in hepatocytes. By contrast, AMPKα2 activity was not modified. Consequently, RSV induced the phosphorylation of ACC by AMPK, rendering ACC inactive, which translates into a decrease in lipid synthesis rates (Figure 2). This fact inhibited high-glucose–induced accumulation of TG in HepG2 cells [23].

Later, Hou et al. [18] studied the mechanisms underlying the effects of RSV on lipid accumulation in HepG2 hepatocytes also exposed to a high concentration of glucose. These authors previously had demonstrated that the dysfunction of hepatic AMPK induced by hyperglycemia represents a key mechanism for hepatic lipid accumulation [23]. The results obtained indicated that RSV prevented the impairment of AMPK phosphorylation and that of its downstream target ACC, the elevation in fatty acid synthase (FAS) expression, and, therefore, the lipid accumulation in human HepG2 hepatocytes. All of these effects were mediated by increased SIRT1 activity, LKB1 phosphorylation, and AMPK activity. In order to confirm these results, the authors performed further experimental procedures. After several experimental approaches, they observed that the positive effects of RSV on AMPK phosphorylation were largely abolished by pharmacological and genetic inhibition of SIRT1, suggesting that the stimulation of AMPK and lipid-lowering effect of the phenolic compound depended on SIRT1 activity. On the other hand, Hou and coworkers, after an adenoviral overexpression of SIRT1, detected a stimulation of basal AMPK signaling in HepG2 cells. Moreover, LKB1 is required for the activation of AMPK induced by RSV and SIRT1. The authors put forward that SIRT1 functions as a novel upstream regulator for LKB1/AMPK signaling and it plays an essential role in the regulation of hepatocyte lipid metabolism (Figure 2).

In another study reported by Shang et al. [24], fat accumulation was also induced in HepG2 hepatocytes by incubating these cells with large amounts of glucose and insulin in the medium. Moreover, RSV was included in the incubation medium at a final concentration of 0.1%. This phenolic compound prevented TG accumulation. This effect seemed to be due to the activation of AMPK and the down-regulation of *srebf1* and *fasn* gene expression, two important AMPK targets and two key genes that are involved in lipogenesis, a metabolic process that allows hepatocytes to accumulate TG.

In other studies, the accumulation of TG in hepatocytes was induced by incubating cells with fatty acids. Thus, Zhang et al. [28] used HepG2 cells incubated with palmitate, a common approach to inducing steatosis in cell culture, to study the role of autophagy in the beneficial effect of RSV on hepatocyte TG accumulation. For this purpose, they incubated these cells with palmitate at 0.1–0.5 mM for 24 h, which were then treated with or without a series of RSV concentrations (10–100 µM) for a further 24 h, or with or without RSV (40 µM) for different time intervals (6, 2, and 48 h). The optimal condition for inducing steatosis was HepG2 incubation with palmitate (0.2 mM) for 24 h. RSV reduced intracellular lipid content induced by palmitate in a dose-dependent manner. RSV also induced autophagy through the up-regulation of LC3-II and the degradation of SQSTM1, as compared with cells treated with palmitate alone, in which autophagy was inhibited by 3-MA (an inhibitor of the early stages) pre-treatment.

The decreased hepatic lipid content stimulated by RSV was attenuated in the presence of autophagy inhibitors (3-MA, BafA1, or CQ). In addition, RSV increased SIRT1 expression in palmitic-stimulated-HepG2 cells in dose- and time-dependent manners. They also validated that the intracellular lipid content reduction induced by RSV was reversed by EX-527 (an inhibitor of SIRT1) or SIRT1 siRNA in palmitic-stimulated HepG2 cells.

By using primary mice hepatocytes, instead of a cell line culture, Tian et al. [29] carried out an experiment where cells were incubated with different fatty acid concentrations, RSV (50 and 100 μM), nicotinamide (a SIRT1 inhibitor, 10 mM), and compound C (an AMPKα inhibitor, 10 mM). Hepatocyte TG content, phosphorylation levels of IκBα and NF-κB p65, as well as gene expression of *tnf*-α, *il-6*, and *il-1b* were higher in fatty acid-treated hepatocytes than in control cells. Phosphorylation of IκBα and NF-κB p65 and the inflammatory markers levels were greater in cells that were treated with fatty acids, RSV and Compound C than in those treated with fatty acids and RSV, indicating AMPK and SIRT1 inhibition can avoid RSV induced improvements. In addition, RSV reversed the reduction in AMPK phosphorylation and SIRT1 protein expression (Figure 2) induced by fatty acids. Taken as a whole, these results show that RSV inhibits the NF-κB inflammatory pathway via AMPK-SIRT1 pathway.

Tang et al. [27] combined incubation with fatty acid and the addition of ethanol to the medium to induce TG accumulation. Their experimental model consisted of HepG2 cells subjected to 48 h culture with 100 μM oleic acid + 87 mM alcohol (O + A) plus the respective treatments with RSV and/or the AMPK activator AICAR or the AMPK inhibitor compound C. The experimental groups used were control, O + A, O + A-RSV (RSV 5, 15, 45, 135 μM), O + A-AICAR (0.5 mM AICAR), O + A-Compound C-RSV (3 µM Compound C and RSV 45 µM). After 48 h treatment, Oil Red O staining showed that the combination of oleic acid and alcohol increased lipid accumulation. That effect was partially reversed by RSV 15, 45, and 135 µM, but not by RSV 5 µM. Similar results were appreciated when TG contents were measured. All RSV doses, except 5 and 15 µM, reversed the effect induced by fat and alcohol. To elucidate the possible mechanisms implicated in the delipidating effect of the polyphenol, 45 μM of RSV were used in combination with AICAR or Compound C. The AMPK phosphorylation ratio was lower in cells that were supplemented by oleic acid and alcohol as compared to control group. The decreased AMPK phosphorylation ratio induced by O + A was reversed when AICAR was added to cultured cells. Finally, RSV stimulated AMPK in a similar manner to AICAR. By the same token, pACC levels were lower in the O + A group than in the control group. At the same time, these levels were normalized by AICAR and RSV, while in the case of the AMPK inhibitor intermediate values were observed. Greater protein expression of sterol regulatory element binding protein 1 (SREBP-1c) and lipin1 were also found in the O + A group when compared with the control group. Again, RSV and AICAR reversed that effect, while the AMPK inhibitor increased both protein expressions, inhibiting the effects that were induced by the polyphenol treatment. All of these results indicated that RSV was preventing hepatocyte lipid accumulation via AMPK-Lipin1.

Finally, a different model was used by Gao et al. [25]. These authors examined the activity of RSV on the suppression of fat accumulation induced by a liver X receptor (LXR) activator. Due to the fact that *srebf1* and *chrebp*, the two master genes that are responsible for de novo lipogenesis are LXR target genes, the activation of the latter leads to hepatic fat accumulation [31,32]. For this purpose, murine Hepa 1–6 hepatocytes (CRL-1830) were incubated with T0901317 (an agonist and thus activator of LXR, 1 μM) with or without RSV (40 μM), and compound C (10 μM) for 24 h. The authors observed that RSV suppressed the hepatocyte lipid droplet increase induced by the LXR agonist (Nile Red staining), while this effect was greatly repressed by Compound C. These results demonstrated that the effect of RSV was indeed mediated by AMPK (Figure 2). RSV prevented the hepatocyte lipid droplet increase induced by T0901317, without reversing the increase in mRNA levels of *srebf1*, *chrebp*, *acc*, and *fasn* induced by the LXR activator.

The effects of RSV derivatives have also been addressed in the literature. Choi et al. [26] worked with SY-102, a synthesized derivative of RSV, which has lower cytotoxicity than the parent compound, but similar potency. In this study, H4IIEC3 rat hepatoma cells were treated with a FFA mixture (0.5 mM, 2:1 oleic acid and palmitic acid) with or without RSV or its derivative (0, 3.3, 10, 30, or 50 µM) for 24 h. Moreover, they also used T0901317 instead of the FFA mixture, in order to activate SREBP-1 expression in cells. By these means, the involvement of the inhibition of this transcriptional factor in the therapeutic effects of RSV and its derivatives could be investigated. The authors observed that T0901317 treatment induced SREBP-1 maturation, and that RSV (30 µM) and SY-102 (10 µM) addition inhibited this process in a dose-dependent manner. Consistent with these results, they also observed that SY-102 reduced *fasn* gene expression induced by T0901317.

Additionally, they examined whether inhibition of AMPK blocked the effect of SY-102 on the T0901317-mediated SREBP-1 induction. Compound C reversed the inhibitory effect of SY-102 on *srebf1* and *fasn* expression, confirming that SY-102 inhibits the maturation and transcriptional activation of SREBP-1 via AMPK/LXR. Based on these data, the authors conclude that RSV and SY-102 are effective in reducing cell lipid accumulation by inhibiting LXR agonist induced SREBP-1 activation, and thus, reducing the expression of key genes in de novo lipogenesis (*fasn*). Moreover, while using AMPK inhibitors, the authors demonstrated the involvement of this kinase in the effects mentioned.

As far as in vitro experiments are concerned, primary mice hepatocytes were cultured with different non-esterified fatty acid (NEFA) concentrations, RSV (50 and 100 μM), nicotinamide (SIRT1 inhibitor, 10 mM), and compound C (10 mM). TG content, phosphorylation levels of IκBα and NF-κB p65 as well as gene expressions of *tnf-a*, *il-6*, and *il-1b* were higher in NEFA-treated hepatocytes than in the controls. RSV supplementation reversed NEFAs effect in a dose-dependent manner. NEFA reduced AMPK phosphorylation and SIRT1 protein expression, whereas RSV increased it. The phosphorylation of IκBα and NF-κB p65 and the levels of inflammatory markers were greater in the cells treated with NEFA+RSV+Compound C than in the cells that were treated with NEFA+RSV, indicating that the inhibition of AMPK and SIRT1 can avoid RSV mediated improvements (Figure 2). Based on these results, it could be considered that the inhibition of the NF-κB inflammatory pathway produced by RSV occurs through the AMPK-SIRT1 pathway.

All of these studies demonstrate that RSV is effective in preventing TG accumulation in different hepatocyte models by activating AMPK, due to its phosphorylation (Figure 2). According to the experimental designs used, this ability takes place under different metabolic conditions. Furthermore, depending on the used experimental conditions, the beneficial effects of RSV occurs in a dose range of 15 and 135 μM. Moreover, this steatosis preventive effect has also been demonstrated by some RSV derivatives, such as SY-102 and Z-TMS.

## 3. In Vivo Studies on Hepatic Steatosis Prevention

Studies that were conducted to analyze the preventive effect of RSV on hepatic steatosis development have been carried out in mice or rats, by administering the phenolic compound at the same time as the diet that induces TG accumulation in liver (Table 2). There are also studies in the literature focused on other types of models, such as animals showing alcohol steatohepatitis or even steatosis induced by administering chemical products.

The first study devoted to analyzing the effects of RSV on liver steatosis induced by diet, as well as the involvement of AMPK, was carried out by Baur et al. [33]. In that study, the authors used middle-aged (one-year-old) male C57BL/6NIA mice fed either a standard diet (SD) or an equivalent high-calorie diet (60% of calories from fat, HC) supplemented or not with RSV at a dose of 22.4 mg/kg/day (0.04%; HCR) for six months. At 18 months of age, the high-calorie diet greatly increased the size and weight of livers, which was associated with fatty liver, whereas RSV prevented these alterations. Indeed, in the histological examination of liver sections, they observed a loss of cellular integrity and the accumulation of large lipid droplets in the livers of the HC but not the HCR group. Searching for a mechanistic explanation to this effect, the authors demonstrated that RSV-treated mice showed a strong tendency towards inducing phosphorylation of AMPK, as well as phosphorylation of ACC at Ser79, thereby decreasing its activity, and decreased expression of *fasn* gene [33].

Moreover, the livers of the RSV-treated mice exhibited considerably more mitochondria than those of mice from HC controls and were not significantly different from those of the SD group. Due to the lack of tissue availability, the authors performed a parallel experiment in the same cohort of animals, but supplemented for six weeks with 186 mg/kg/day. In this case, the authors also observed increased mitochondrial biogenesis in liver through the deacetylation of PGC-1α (peroxisome proliferator activated receptor gamma, coactivator 1α), a master regulator of this process. It has been reported that AMPK promotes PGC-1α activation with the involvement of SIRT1 [34], and in turn, the acetylation status of PGC-1α is considered a marker of SIRT1 activity in vivo. Importantly, since Baur et al. did not find changes in SIRT1 protein levels in RSV-treated mice. As a result of the increase in mitochondrial number and PGC-1α activation, these authors suggested that SIRT1 enzymatic activity was enhanced by RSV [33].

In the work reported by Tian et al. [29], the authors not only evaluated the effect of RSV on TG accumulation, but they also extended the study to inflammatory markers. For this purpose, they fed C57BL/6 mice a high-fat (HF) diet (60% fat) for 60 days, treated or not with RSV (30 mg/kg body weight/day). Resveratrol-treated mice showed decreased liver weight and reduced hepatic TG content to an intermediate level between that found in mice fed the HF diet and those observed in a control group fed a standard diet. Regarding plasmatic levels, reduced levels of transaminases were also observed. Moreover, RSV supplementation avoided the inactivation of AMPKα induced by the HF diet, as shown by the increased pAMPKα/AMPKα ratio. Moreover, SIRT1 expression followed the same trend. As far as inflammation markers are concerned, phosphorylated IkBα and NF-kB p65, IL-1β, Il-6, and TNF-α were overexpressed in HF diet-fed mice. However, this effect was avoided by RSV.

Other studies reported in the literature have been performed in rats. In a study that was carried out by our group we fed Sprague-Dawley rats a high-fat high-sucrose diet supplemented or not with the amount of RSV needed to achieve a dose of were 30 mg/kg body weight/day for six weeks. RSV did not reduce liver weight or serum ALT and AST concentrations, but did prevent increased hepatic fat infiltration. To elucidate the mechanisms involved in the delipidating effect we analyzed some the activity of enzymes involved in lipogenesis and fatty acid oxidation, as well as other mitochondrial markers. The polyphenol limited hepatic lipogenesis as shown by the reduction in ACC activity, although no changes were observed in FAS, malic enzyme (ME), and glucose-6P-dehydrogenase (G6PDH) activities, or in gene expression of *srebf1*, a transcriptional factor that regulates FAS. Moreover, RSV increased carnitine-palmitoyl-1a (CPT1a) and acyl-CoA oxidase (ACO) activities and activated PGC-1α, indicating an increase in mitochondrial and peroxisomal fatty acid oxidation, even though the gene expression of *pparγ* and *pgc1α* was not modified. *Tfam* and *cox2* expression, two indicators of mitochondrial genesis and oxidative phosphorylation respectively, remained unaltered by RSV treatment. In view of these results, we proposed that increased CPT1a activity was not due to increased mitochondria number. As far as AMPK was concerned, in this study RSV increased its activity since phosphorylated AMPK/total AMPK protein ratio was augmented. For that reason we proposed the potential involvement of the AMPK in RSV reducing fatty liver infiltration effect [36].

The influence of RSV on imprinting has also been analyzed. This term refers to the fact that nutritional and other environmental factors in early life have a profound influence on lifelong health. Tanaka et al. [38] carried out a study devoted to investigating whether maternal RSV administration affected lipogenesis in male offspring, by means of lactation, once they reached adult age. If this was the case, the authors also wanted to determine the mechanisms that are involved in the effects observed. For this purpose, pregnant Wistar rats were divided into two groups fed a control diet with RSV or not. The group supplemented with the polyphenol received a RSV dose of 20 mg/kg body weight/day by gavage during lactation (three weeks), while the control group received the vehicle. Once the lactation period was completed, six male pups for each group (control and RSV-treated group) were fed a standard diet. Animals were sacrificed when they were 36 week-old. When liver sections of the animals in both groups were stained (hematoxylin-eosin), the lipid droplet amount found in the samples of the RSV group was lower than that found in the control group. No information was reported regarding liver weight or liver TG content. Using the western-blot technique, a significant increase in the pAMPK/AMPK ratio was found in the RSV-treated group. In the same way, greater SIRT1 protein expression was also found in the animals of this group when compared with the control group. Moreover, when analyzing ACC and FAS, the two key enzymes of lipogenesis, lower activities, and protein expressions were found. Finally, the authors also measured the proteolytic processing of SREBP-1c, which is the transcriptional factor that regulates *acc* and *fasn* gene expression. In this case, a significantly lower SREBP-1c active form/precursor form ratio was found in the RSV group, suggesting that, in this case, the activation of this transcriptional factor was lower. Based on the results obtained, the authors concluded that maternal RSV intake during lactation effectively down-regulated hepatic lipogenesis in adult male rats. Moreover, the authors suggested that such effects could be due to epigenetic modifications induced by the polyphenol. The lipid lowering effects that were observed in the animals from the RSV group seemed to be mediated, at least in part, by the greater AMPK-SIRT1 axis activation induced by the compound (Figure 2).

In addition to non-alcoholic liver steatosis, hepatic TG accumulation can also be induced in humans by chronic high intake of ethanol. Thus, animal models imitating this situation have been developed, and the effectiveness of RSV on ethanol-induced steatosis has been addressed. In this context, Ajmo et al. [35] performed a study in mice that were fed a low-fat diet supplemented with ethanol (29% of total calories). Mice were divided into three experimental groups: (1) low-fat diet plus ethanol, (2) low-fat diet plus ethanol and 200 mg/kg body weight RSV (E+R200), and (3) low-fat diet plus ethanol and 400 mg/kg body weight/day RSV (E+R400). RSV was added to the diets during the last two weeks of the study.

RSV treatment increased SIRT1 expression levels and stimulated AMPK activity in livers of ethanol-fed mice. The protective action of RSV was in whole or in part mediated through the up-regulation of a SIRT1-AMPK signaling system (Figure 2) in the livers of ethanol-fed mice. The final conclusion was that RSV treatment led to reduced lipid synthesis and increased rates of fatty acid oxidation, thus preventing alcoholic liver steatosis and so it might represent a promising agent for the prevention and treatment of human alcoholic fatty liver disease.

Finally, Gao et al. [25] examined the effect of RSV on the suppression of fat accumulation induced by the activation of LXR, a transcriptional factor that facilitates TG accumulation through a compound known as T0901317, as they carried out with murine Hepa 1–6 hepatocytes. They divided C57BL/6 mice into three groups: the carrier solution-treated control group, a group treated intraperitoneally with T0901317 (5 mg/kg body weight/day), a group treated intraperitoneally with T0901317 (5 mg/kg body weight/day) and orally with RSV (200 mg/kg body weight/day) for a short period (five days). In the T0901317-treated group the liver became bigger, with higher lipid and number of bright red spots in tissue sections, analyzed by staining in tissue sections, and increased serum TG and cholesterol levels. RSV prevented liver size increase and reduced hepatic TG amounts. Moreover, it completely blocked serum TG and cholesterol levels elevation. By contrast, transaminase levels were not affected by treatments.

When gene expression analysis was carried out, it was observed that T0901317 markedly increased the expression of *acc*, *srebf1* and *chreb*, but RSV did not have any impact on these genes. However, treating with RSV alone, *acc*, *srebf1,* and *chreb* gene expression decreased. Immunohistochemistry and western analysis revealed that mice treated at the same time with T0901317 and RSV showed a marked increase in pAMPK (Thr-172) and pACC (Ser-79, inactivation) as compared to mice treated with T0901317 alone. The conclusion of this study is that RSV activated AMPK by increasing its phosphorylation at the post-translational level, and consequently reduced carboxylase activity of ACC, thus suppressing lipogenesis and fat accumulation in the liver (Figure 2).

As in the case of in vitro studies, the effects of RSV derivatives have also been analyzed in animal models. Tung et al. carried out a study aimed at analyzing the inhibitory effect of piceatannol, a natural stilbene that is an analog and a metabolite of RSV, on HF diet-induced obesity in C57BL/6 mice [37]. The authors also included in the experimental design a group supplemented with RSV. For this purpose, five week-old male C57BL/6 mice were fed a HFD (45% of the energy as fat) for 18 weeks, alone or supplemented with RSV (0.1% *w*/*w*) or piceatannol (0.1% or 0.25% *w*/*w*). At the end of the experimental period, lower liver weights were found in the two groups supplemented with piceatannol when compared with the HF group, while this parameter remained unchanged in the group receiving RSV. As far as serum transaminases is concerned, aspartate aminotransferase (AST) and alanine aminotransferase (ALT) levels remained unchanged in all of the treated groups when compared with the HF group. The activation of AMPK in the liver, along with the expression of different adipogenic proteins, was assessed by means of western-blot. Greater AMPK phosphorylation (in threonine 172 residue) was found in all of the groups treated (with no difference among them) in comparison with the group fed the HFD alone. This means that RSV and piceatannol significantly enhanced the activation of this kinase. Moreover, a similar phosphorylation pattern was also observed in the case of acetyl CoA carboxylase (ACC) (in serine 79 residue) (Figure 2). In this case, the greater phosphorylation resulted in a lower activation of this lipogenic enzyme. Finally, the authors also found a significant reduction in the protein expression of FAS in the groups in which RSV and piceatannol were administered. Based on those results, it could be stated that, under these experimental conditions, both RSV and piceatannol are effective in inducing the activation of AMPK in the liver of mice that were fed an obesogenic diet. Moreover, a concomitant reduction in the activation or protein expression of key lipogenic proteins (ACC and FAS) was also described. Nevertheless, in these conditions piceatannol seemed to be more effective than RSV in reducing liver lipid content. Although the activation of AMPK may be involved in the effects observed, due to the fact that the activity of enzymes that are involved in fatty acid oxidation (such as CPT1a) was not measured by the authors, no conclusion can be drawn in this respect.

Altogether, these studies confirm the results obtained in in vitro studies using isolated hepatocytes. In fact, when RSV administration takes place at the same time as the cause inducing TG accumulation, this phenolic compound prevents partial or totally this alteration. This beneficial effect has been reported in a very huge range of doses, from 22.4 to 400 mg/kg body weight/day and using experimental periods, from two weeks to six months. As in the case of in vitro studies, it seemed that the activation of AMPK was mediating, at least in part, the hepatic delipidating effect. Moreover, piceatannol, a RSV derivative that shows two hydroxyl groups in the benzene ring, instead of three as in the case of RSV, shows the same effect but it was even more effective that its parent compound.

## 4. In Vivo Studies on Hepatic Steatosis Treatment

There are also studies devoted to analyzing the effect of RSV on previously developed liver steatosis in the literature, in other words, studies that address its therapeutic effects on this hepatic metabolic alteration (Table 3). Shang et al. [24] carried out an experiment by feeding rats HF-diet (59% calorie from lard fat, 21% from protein, 20% from carbohydrate) for six weeks. At the end of this period, half of the HF group was treated orally with RSV (100 mg/kg body weight/day; HR group), and the other half was treated with saline. At the end of the 16th week, all of the animals were sacrificed. Liver histology showed that rats fed the HF diet developed liver steatosis and insulin resistance, which were markedly improved by 10 weeks of RSV administration. In addition, this compound promoted the phosphorylation of AMPK, which in this study suppressed the expression of genes that are related to lipogenesis, thus contributing to the improvement of liver steatosis and insulin resistance. The authors concluded that, by reducing TG accumulation and improving insulin resistance through AMPK activation, RSV could protect the liver from NAFLD.

In another study, Lee et al. [44] fed C57BL/6J mice a standard or a HF diet (45% fat), and then continued the experiment for four additional weeks treating mice with RSV (8 mg/kg body weight/day) or vehicle (control group), administered by using an osmotic pump. The aim of this study was to analyze both the relationship between adiponectin and fetuin-A and whether RSV alters both cytokines and several related factors.

At the end of the treatment, liver weight and hepatic index were reduced by RSV supplementation. Serum TG and ALT were also normalized to control levels, but serum AST, increased by the HF diet, was not re-established by the polyphenol. These data indicate that this polyphenol improved liver function, which is apparently mediated by fetuin-A, a hepatokine that mediates fatty liver-induced cardiometabolic diseases. As occurred in serum, hepatic *fetuin-A* gene expression and protein levels were significantly increased by the HF diet, and RSV re-established control values. Gene expression of *ampk* in liver was reduced by the HF diet and highly increased in RSV-treated mice, to a level even higher than that observed in the controls. In addition, *nf-κb* gene expression, a downstream target of fetuin-A, was higher in HF animals. Taking these results into account, the authors concluded that under their experimental conditions RSV probably improved the steatosis by the AMPK-NF-κB-fetuin-A axis.

Choi et al. [26] who evaluated the effect of SY-102, a synthesized derivative of RSV, on cell cultures also carried out an experiment in ICR mice with this compound. Animals were randomly assigned to six groups: a control group fed a purified diet for five days and the other groups were fed a HF diet (27% safflower oil; 59% fat-derived calories) for five days. During the last two days of the experimental treatment, mice were treated daily with the RSV or the RSV derivative (SY-102) by oral injection (15 and 45 mg/kg body weight/day, respectively). Liver TG content increased by two-fold in the HF group, which decreased back to the control level by treatment with SY-102. This compound also recovered the impaired AMPK phosphorylation caused by the HF, in a dose-dependent manner and prevented the increase *srebf1* and *fasn* mRNA levels induced by HF diet. The authors concluded that SY-102 improved HF-induced fatty liver in mice through the AMPK/LXR pathway.

Zhang et al. [28] determined the role of autophagy in the beneficial effect of RSV on hepatic steatosis. The authors used 129/SvJ mice (eight-week-old) that were fed a HF diet (containing 60% fat) or chow diet (10% fat) for four weeks to induce hepatic steatosis. After this period, HF-fed mice were further divided into two subgroups, which were fed chow diet (HF+chow group) or chow diet containing RSV (0.4%) (HF+RSV group) for four additional weeks. The authors observed that after four weeks of treatment, RSV reduced the weight of HF-fed mice, without significant changes in food intake. The HFD+chow group showed increases in lipid content in the liver compared with the control group, which were markedly attenuated by the addition of RSV (0.4%) (HFD+RSV group) to the diet for a further four weeks. RSV significantly increased SIRT1 activity, the pAMPK and pPRKA (protein kinase A) levels in liver tissues of mice fed with HFD (HFD+RSV group). RSV also increased adenylate cyclase expression and cAMP levels in liver tissues of HFD-fed mice (HFD+RSV group). In conclusion, RSV-induced autophagy in response to hepatic steatosis through the cAMP-PRKA-AMPK-SIRT1 signaling pathway (Figure 2).

Recently, in a study carried out in our group [1], we fed rats a high-fat high-sucrose diet for six weeks. At the end of this period, nine animals were sacrificed in order to determine whether hepatic steatosis had been induced. The liver lipid content of these rats was compared with that of a matched group of rats fed a standard diet for six weeks. Once the development of hepatic steatosis was confirmed, the remaining rats were shifted to a standard diet (STD) supplemented with RSV (30 mg/kg body weight/day) or not for six additional weeks. At the end of the experimental period (12 weeks), significantly lower hepatic TG levels were found in the group treated with RSV. In order to know the mechanisms that are involved in this hepatic lipid lowering effects of RSV, the activities of several enzymes involved in lipid metabolism were assessed. CPT1a and citrate synthase (CS) activities were observed in the group receiving the compound, which points toward increased mitochondrial fatty acid oxidation and density (respectively). Moreover, when measuring the effects of RSV on microsomal triacylglycerol transfer protein (MTP) activity, a greater activation of this enzyme was appreciated when the polyphenol was administered to the animals, suggesting that, in this group, the plasma TG release from the liver was enhanced. The activations of AMPK and ACC, as well as the protein levels of fatty acid transport protein 5 (FATP5) were studied by means of western-blot. RSV significantly increased AMPK phosphorylation (in threonine 172 residue) and thus activation, while decreasing the protein expression of FATP5. In the case of ACC, although no significant differences were found between both groups, a trend towards an increased phosphorylation status (the serine 79 residue) was observed in the animals that were supplemented with RSV. Based on the data obtained, we concluded that RSV was effective in reducing hepatic steatosis that was previously induced by an obesogenic feeding pattern. It could be suggested that the increased AMPK activation found in the group receiving RSV could, to some extent, influence the greater fatty acid oxidation through the reduction of ACC activity, and thus malonyl-CoA production (an inhibitor of CPT1a). 

Kang et al. [41] hypothesized that RSV action on insulin signaling could depend on the metabolic state of cells and that it is tissue specific. Thus, they evaluated the effect of RSV on insulin action in insulin-sensitive tissues in mice fed a HF diet. For that purpose, they treated diet-induced obese C57BL/6N mice with RSV, at a dose of 30 mg/kg body weight/day for two weeks. RSV did not affect body weight in HF-fed mice, but reduced both fasting glucose and insulin levels, suggesting an insulin-sensitizing effect. In order to explain this effect, they assessed insulin signaling pathway and the activation state of AMPK in liver. RSV restored diminished insulin signaling induced by phosphorylation of AKT in Ser473 and Thr308 residues. By contrast with the most common studies in the literature, AMPK was more active in HF mice when compared to control mice and this activation was partially reverted after RSV treatment (not to the level of control mice).

After a histological examination of the liver, fat increase induced by the HF diet was reversed by the short-term RSV treatment. It is important to point out that this effect was independent of body weight change that, as previously indicated, was not significantly reduced after RSV treatment.

In addition to works addressing models of liver steatosis that are associated to diet, other experiments have been performed by using genetically models of obesity or diabetes. Rivera et al. [39] treated obese Zucker rats (Zucker *fa/fa* rats), which show a strong liver steatosis early in life, and their heterozygous lean littermates (Zucker *Fa/Fa*) with RSV at a dose of 10 mg/kg body weight/d for eight weeks. Food intake, body weight, liver weight, as well as hepatic TG and cholesterol content were notably greater in obese rats than in their lean littermates. RSV treatment attenuated the increase in hepatic TG content, by reducing it by half and cholesterol content, which reached lean levels. These reductions seemed to be due to AMPK activation by phosphorylation of its Thr172 residue and the consequent ACC inactivation by phosphorylation (Figure 2).

Do et al. [40] analyzed the effects of RSV on steatosis in a model of diabetic mice (*db/db* mice). In this study, they distributed animals into four experimental groups: control group, a group treated with 0.001% (*w*/*w*) of the hypoglycemic agent rosiglitazone, a group treated with 0.005% (*w*/*w*) RSV, and a group treated with 0.02% (*w*/*w*) RSV for six weeks. This review will focus only on the effects of RSV. Regarding hepatic lipid content, only the highest dose of this phenolic compound reduced TG when mice were compared with the controls, but it did not affect hepatic cholesterol levels. With regard to protein levels, no quantification figures appear in the publication and only protein bands can be observed; consequently, only data commented by authors can be discussed. Phosphorylated ACC, measured as an indicator of de novo lipogenesis, was reduced by RSV and the protein expression was lower with the highest dose of RSV compared with the lowest one. Moreover, 0.02% RSV increased *srebf1* gene and PPARα protein expression, although no differences were observed in *pparα* gene expression. As far as fatty acid β-oxidation is concerned, RSV increased UCP2 protein expression, which is regulated by PPARα. When they determined AMPK activation, they observed higher phosphorylated AMPK protein levels in RSV-treated mice compared with control mice; this activation was higher at a dose of 0.005% of RSV. Authors concluded that RSV was able to reduce liver steatosis in their experimental model of type 2 diabetes by activating AMPK signaling.

Lin et al. [42] studied the effects of RSV on hepatitis B virus (HBV) associated fatty livers at an early stage of pathogenesis. The work was carried out in C57BL/6 transgenic mice, which spontaneously develop hepatocellular carcinoma at the age of 13–16 months because they express the HBV X protein, specifically in hepatocytes, and in wild type mice. Animals received orally RSV at a dose of 30 mg/kg body weight/day and they were sacrificed at 2, 3, 7, and 14 days. In the transgenic animals, microsteatosis, pleomorphic and bizarre nuclei, ballooning and abnormal arrangements of the sinusoids were observed at 4–6 weeks. The polyphenol mitigated liver impairment and reverted histopathological alterations. After 14 days of treatment, RSV-treated mice had no fatty liver and serum ALT was significantly reduced. Regarding the mechanism of action, RSV reduced *srebf1* and *lxrα* but no *lxrβ* gene expressions on day 2. The ratio of phosphorylated AMPK/total AMPK, as representative of its activation, was increased on day 3, and the gene expression of *pparγ* and *acc* was decreased. Moreover, SIRT1 was activated, its protein expression increased and *fasn* gene expression decreased on day 7 of treatment with RSV. Finally, no changes were observed in phosphorylated AKT/total AKT ratio, as representative of AKT activation, and *scd1* gene expression but no changes were observed. When considering these results, it can be observed that the reduction in hepatic lipid content was, at least in part, due to a reduction in the lipogenic pathway; probably the down-regulation of srebf1 decreased *acc* and *fasn* gene expression, but the reduction in *srebf1* was not due to the AMPK pathway.

Finally, Zhu et al. [43] investigated the potential benefits of RSV on the amelioration of oxidative stress and hepatic steatosis in a model of genetic obesity, the KKAy mouse. In this study, C57BL/6J mice were used as controls. The KKAy mice were randomly divided into three groups: a standard group that was fed a chow diet (KKAy group), a group that was treated with a low dose of RSV (KKAy + Low RSV), and a group treated with a high dose of RSV (KKAy + High RSV). The control and KKAy groups were fed a standard AIN93G diet, while the Low RSV and High RSV groups were fed a standard AIN93G diet supplemented with RSV at doses of 2 and 4 g/kg diet, respectively, for 12 weeks. They observed that in the KKAy group body weight and hepatic index were higher in comparison to values observed in C57BL/6J mice, but they were reversed in the KKAy+high RSV group. Serum levels of FFA and malonaldehyde (MDA) in KKAy mice were higher than those in C57BL/6J mice, while the superoxide dismutase (SOD) level was decreased. MDA levels in the RSV treatment group were significantly decreased when compared with the KKAy group. ROS level was decreased by RSV, while levels of glutathione (GSH), glutathione peroxidase (GPx), and SOD were increased.

The authors also carried out a histological study of liver, where they noticed fresh bright red liver tissue in C57BL/6J mice, but in non-treated KKAy mice. The degree of hepatic steatosis evaluated by Oil Red was significantly alleviated by RSV. Similarly, the liver TG level was reduced in RSV-treated mice as compared with non-treated KKAy mice, whereas there was an insignificant change in TC level. *hsl* and *atgl* mRNA levels were decreased in KKAy mice compared with C57BL/6J, and this decrease was reversed by high RSV treatment. Moreover, pHSL protein was highly expressed in both the low and high RSV treatment groups and SIRT1, pAMPK α, pFOXO1 and FOXO1 (cytoplasm) in KKAy mice was reversed by RSV treatment, when compared with C57BL/6J mice. The authors concluded that RSV ameliorated hepatic steatosis inducing up-regulation of SIRT1 and AMPK.

The studies described demonstrate that, in addition to its preventive effect, RSV is also able to reduce hepatic TG accumulation, independently of the steatosis model used, thus representing a potential interesting approach to treat liver lipid alteration. As well as in the case of steatosis prevention, the improvement that was observed in steatosis previously developed is also due, at least in part, to increased AMPK activation.

## Figures and Tables

**Table 1:** In vitro studies conducted to test the effect of resveratrol in liver cells.

Reference	Cell Line	Experimental Design	Effect of Resveratrol	Mechanism of Action
[23]	HepG2	24 h culture with RSV (10 µmol/L) 1 h culture with RSV (50 µmol/L) (AMPKα1 activity determination)	Prevention of high-glucose-induced lipid accumulation (in HepG2 cells)	↑ AMPK (Thr-172) and ACC (Ser-79) phosphorylation (10 µmol/L RSV) ↑ AMPKα1 activity
[18]	HepG2 HEK293	HepG2 24 h culture with RSV (1–100 μM) HEK293 cells pretreated with splitomicin (100 μM) for 24 h and incubated with RSV (50 μM) for an additional 1 h	↓ High glucose-induced TG accumulation: RSV (10–50 μM)	↑ SIRT1 activity (dose dependent (10–100 μM RSV) ↓ FAS protein expression ↑ AMPKα phosphorylation (Thr172) ↑ ACC phosphorylation (Ser79)
[24]	HepG2	6 or 24 h culture with 10, 25, and 50 μM of RSV	↓ TG accumulation	↑ AMPKα phosphorylation (Thr172) ↓ *srebf1* and *fasn* gene expressions
[25]	Hepa 1–6 cell line (murine hepatocytes)	24 h culture with T0901317 (1 μM, LXR activator: ↑ liver fat accumulation) + RSV (40 μM), with or without compound C (10 μM, AMPK inhibitor)	↓ T0901317-induced fat accumulation	↓ T0901317-induced fat accumulation (via AMPK activation)
[26]	H4IIEC3 rat hepatoma cells	24 h culture with FFA [oleic acid and palmitic acid 2:1, 0.5 mM] or T0901317 (10 μM, LXR activator) + RSV or SY-102 (RSV derivative, 3.3–50 μM)	↓ FFA-induced lipid accumulation by RSV (50 mΜ) and SY-102 (30 μM) ↓ T0901317-induced SREBP-1 maturation by RSV (30 μM) and SY-102 (10 μM)	↓ *srebf1* and *fasn* gene expression by SY-102 (50 μM) via AMPK/LXR pathway
[27]	HepG2	48 h treatment Control, oleic acid + alcohol (O + A), O + A-RSV (5, 15, 45, 13 5 µM), O + A-AICAR-RSV 45 µM, O + A-Compound C-RSV 45 µM	↓ Lipid accumulation (15, 45, 135 μM) ↓ Hepatocyte TG content (45 and 135 μM) Attenuated hepatic steatosis	↑ AMPKα phosphorylation * ↑ ACC phosphorylation * ↓ SREBP1c and lipin protein expression
[28]	HepG2	24 h culture with 0.2 mM palmitate Additional 24 h treated with RSV (20–80 μM) Also exposed to 3-MA autophagy inhibitor for 1 h or siRNAs before the addition of RSV	↓ Lipid content	↑ LC3-II protein expression and SQSTM1 protein degradation (3-MA pre-treatment inhibited this effect) ↑ SIRT1 protein expression/activity and cyclic AMP levels ↑ AMPK (Thr-172) and PRKA (Ser-96) phosphorylation
[29]	Primary hepatocytes from C57BL/6 mice	Treatment with NEFA, NEFA + RSV (50 and 100 μM), NEFA + Nicotinamide, NEFA + Compound C, NEFA + RSV + Nicotinamide, NEFA + RSV + Compound C Treatment length not specified	↓ NEFA increased expression of several inflammatory markers	↑ AMPK phosphorylation * ↑ *sirt1* gene and SIRT1 protein expressions ↓ phosphorylation IκBα and NF-κB p65 ↓ *il-1β*, *il-6*, and *tnf-α* gene expression

3-MA: 3-Methyladenine, ACC: acetyl CoA carboxylase, AICAR: 5-Aminoimidazole-4-carboxamide ribonucleotide, AMPK: 5′AMP-activated protein kinase, FAS: fatty acid synthase, FFA: free fatty acid, IκBα: inhibitor of kappa B, IL-1β: interleukin 1 beta, IL-6: interleukin 6, LC3-II: microtubule-associated protein1 light chain 3, LXR: liver x receptor, NEFA: non-esterified fatty acid, NF-κB: nuclear factor-κB, PRKA: monophosphate-activated protein kinase, RSV: resveratrol, SIRT1: silent information regulator 1, SREBF1: sterol regulatory element binding transcription factor 1, SREBP1: sterol regulatory element binding transcription factor 1, SQSTM1: sequestosome 1, TG: triglyceride, TNF-α: tumor necrosis factor α. ↑: increased, ↓: decreased. * The measured phosphorylation residue is not mentioned in the article.

**Table 2:** Preclinical studies conducted in vivo to test the preventive effect of resveratrol on hepatic steatosis.

Reference	Animal Model	Experimental Design	Effect of Resveratrol	Mechanism of Action
[33]	One-year-old male C57BL/6NIA mice	HFD (60% of calories as fat) RSV dose: 22.4 mg/kg bw/day Length: 6 months	Fatty liver development prevention (organ size) Prevention of cellular integrity loss and large lipid droplet accumulation	↑AMPK phosphorylation (Thr172) ↑ACC phosphorylation (Ser79)
[35]	6–8 week-old male C57BL/6J mice	LFD (10% of calories as fat) RSV dose: 200 and 400 mg/kg bw/day 3 groups: LF diet+ethanol, LF diet + ethanol + RSV200, LF diet + ethanol + RSV400 Length: 2 weeks	Prevention of liver weight, liver lipid droplets, hepatic TG content and serum ALT level increase	↑*sirt1* gene and SIRT1 protein expressions ↑ AMPKα and β phosphorylation * ↑ total AMPK levels ↑ ACC phosphorylation * ↓ SREBP1c protein expression ↓ *fasn*, *gpat1*, *scd1*, *accα*, *me* ↑*acox1*, *mcad* and *cpt1a* gene expression ↓ *pparγ* gene expression
[36]	Male Sprague Dawley rats	Obesogenic diet (45% of calories as fat) RSV dose: 30 mg/kg bw/day Length: 6 weeks	↓ Hepatic fat content	↑ AMPK phosphorylation (Thr172) ↑ ACC phosphorylation (Ser79)
[25]	Male C57BL/6 mice induced by LXR receptor	Groups: Control, T0901317 (LXR activator) and T0901317+ RSV RSV dose: 200 mg/kg bw/day Length: 5 days	Prevention of the increase in liver size, fat accumulation and TG content (induced by LXR activator)	↑ AMPK phosphorylation (Thr172) ↑ACC phosphorylation (Ser79) ↓ *srebf1, chrebp* and *acc* expression (RSV alone)
[29]	4 week-old C57BL/6 mice	HFD (60% of calories as fat) RSV dose: 30 mg/kg bw/day Length: 60 day	↓ Liver weight ↓ GGT, AST, ALT, ALP, LDH plasma levels ↓ IL-1β, IL-6, and TNF-α plasma levels	↑ AMPK phosphorylation (Thr172) ↑ SIRT1 protein expression ↓ IkBα and NF-kB p65 phosphorylation * ↓ *il-1β, il-6*, and *tnf-α* gene expression
[37]	5 week-old male C57BL/6 mice	HFD (45% of energy as fat) RSV dose: 0.1% resveratrol (*w*/*w*) Length: 18 weeks	No changes in liver weight and serum AST and ALT levels	↑ AMPK phosphorylation (Thr172) ↑ACC phosphorylation (Ser79) ↓ FAS protein expression ↓ Hepatic adipogenic protein expression
[38]	Pups from female Wistar rats	Control diet RSV dose: 20 mg/kg bw/day Length: 3 weeks (lactation period)	↓ Hepatic lipid accumulation	↑ AMPK phosphorylation (Ser403) ↑ SIRT1 protein expression ↓ Active/precursor SREBP-1c protein ratio ↓ ACC protein expression ↓ FAS protein expression ↓Hepatic adipogenic protein expression

ACC: acetyl CoA carboxylase, ACOX1: acyl-Coenzyme A oxidase 1, ALP: alkaline phosphatase, ALT: alanine aminotransferase, AMPK: 5′AMP-activated protein kinase, AST: aspartate aminotransferase, bw: body weight, chrebp: carbohydrate response element binding protein, CPT1a: carnitine palmitoyltransferase 1a, FAS: fatty acid synthase, GGT: gamma glutamil transpeptidase, GPAT1: glycerol-3-phosphate acyltransferase 1, HFD: High-fat diet, IL-1β: interleukine-1β, IL-6: interleukine, LDH: lactate dehydrogenase, LFD: low fat diet, LXR: liver x receptor, MCAD: mitochondrial medium-chain acyl-CoA dehydrogenase, ME: malic enzyme, pIkBα: phospho-inhibitory subunit of NF-KBα, NF-KBβ p65: nuclear factor kappa-light-chain-enhancer of activated B cells subunit p65, PPARγ: peroxisome proliferator-activated receptor γ, SCD1: stearoyl-Coenzyme A desaturase 1, RSV: resveratrol, SIRT1: silent information regulator 1, SREBF1: sterol regulatory element binding transcription factor 1, SREBP-1c: sterol regulatory element binding protein-1c, TG: triglyceride, TNF-α: tumor necrosis factor-α. ↑: increased, ↓: decreased. * The measured phosphorylation residue is not mentioned in the article.

**Table 3:** Preclinical studies conducted in vivo to test the therapeutic effect of resveratrol on hepatic steatosis.

Reference	Animal Model	Experimental Design	Effect of Resveratrol	Mechanism of Action
[24]	Male Wistar rats (180–200 g)	Acute treatment: Fed stated rats RSV dose: 100 mg/kg bw/day Length: 4 h	↓ Hepatic fat content	↑ AMPK phosphorylation (Thr172) ↓ *srebf1* and *fasn* gene expressions
		Chronic treatment: High-fat diet (59% of calories as fat) RSV dose: 100 mg/kg bw/day Length: 10 weeks		
[39]	Obese male Zucker rats and lean heterozygous littermates	STD RSV dose: 10 mg/kg bw/day Length: 8 weeks	No change in liver weight ↓ Liver TG and cholesterol content	↑ AMPK phosphorylation (Thr172) ↑ ACC phosphorylation *
[40]	4 week-old male C57BL/KsJ-db/db mice	RSV dose: 0.005% and 0.02% (*w*/*w*) Length: 6 weeks	↓ Hepatic fat content (only in 0.02% RSV group)	↓ ACC phosphorylation * ↑ *srebf1* gene expression (0.02% RSV) ↑ PPARα protein expression (0.02% RSV) ↑ UCP2 protein expression ↑ AMPK phosphorylation *
[41]	5 week-old male C57BL/6N mice	HFD RSV dose: 30 mg/kg bw/day Length: 2 weeks	↓ Hepatic fat content	↑ AKT phosphorylation (Ser473 and Thr308) ↓ AMPKα phosphorylation (Thr172)
[42]	4 week-old male C57BL/6 mice expressing HBV X protein	RSV dose: 30 mg/kg bw/day Length: 2, 3, 7, and 14 days	Histopatology alteration reversion ↓ Serum ALT levels	↓ *srebf1* and *lxrα* gene expressions (from day 2 in advance) ↑ AMPK phosphorylation (Thr172) (from day 3 in advance) ↓ *pparγ* and *acc* gene expressions. ↑ SIRT1 protein expression and activity (from day 7 in advance) ↓ *fasn* gene expression
[26]	Male ICR mice (20–25 g)	HFD. RSV dose: 15 or 45 mg/kg bw/day. (same doses of SY-102, a RSV derivative). Length: 2 days	↓ Hepatic TG levels (by SY-102 and RSV)	↑ AMPK phosphorylation (Thr172) (by SY-102) ↓ *srebf1* and *fasn* mRNA levels (by SY-102 and RSV)
[43]	8 week-old male KKAy mice (genetic model of obesity) 8 week-old male C57BL/6J mice (control)	Chow diet (AIN93G) RSV dose: 2 or 4 g/kg diet Length: 12 weeks	↓ Hepatic fat content (Oil Red) and TG levels Hepatic steatosis attenuation (histological study) ↓ MDA levels	↑ AMPK phosphorylation (Thr172) ↑ SIRT1 protein expression ↑ FOXO1 phosphorylation (Thr24) ↓ ROS levels ↑ GSH levels, GPx and SOD activities ↑ *hsl* gene expression and HSL phosphorylation (Ser660) ↑ *atgl* gene expression and ATGL protein expression
[44]	6 week-old male C57BL/6J mice	HFD RSV dose: 8 mg/kg bw/day Length: 4 weeks	↓ Liver weight ↓ Plasma levels of Fetuin-A and ALT ↓ Hepatic index	↑ *ampk* gene expression ↓ *fetuin-A* gene expression ↓ Fetuin-A protein expression ↓ *nfκβ* gene expression
[28]	8 week-old 129/SvJ mice (male)	HFD (60% of energy as fat) RSV dose: 0.4% (*w*/*w*) Length: 8 weeks	↓ Hepatic fat content	↑ cyclic AMP levels ↑ PRKA phosphorylation (Ser96) ↑ AMPK phosphorylation (Thr172) ↑ SIRT1 protein expression
[1]	6 week-old male Wistar rats	HFHS RSV dose: 30 mg/kg bw/day Length: 6 weeks	↓ Hepatic TG content ↑ Plasma TG release (from liver) ↓ Liver fatty acid uptake	↑ AMPK phosphorylation (Thr172) ↑ CPT1a activity ↑ CS activity ↑ MTP activity ↓ FATP5 protein expression

ACC: acetyl CoA carboxylase, ALT: alanine aminotransferase, AMP: adenosine monophosphate, AMPK: 5′AMP-activated protein kinase, ATGL: adipose triglyceride lipase, ATP: adenosine triphosphate, bw: body weight, CPT1a: carnitine palmitoyltransferase 1a, CS: citrate synthase, FAS: fatty acid synthase, FATP5: fatty acid transport protein 5, FOXO 1: forkhead box protein O1, GPx: glutathione peroxidase, GSH: reduced glutathione, HFD: High-fat diet, HFHS: High-fat high-sucrose diet, HSL: hormone sensitive lipase, LXR: liver x receptor, MDA: malonaldehide, MTP: microsomal triglyceride transfer protein, NF-KBβ p65: nuclear factor kappa-light-chain-enhancer of activated B cells, PPARγ: peroxisome proliferator-activated receptor γ, PPARα: peroxisome proliferator-activated receptor α, PRKA: protein kinase A, ROS: reactive oxygen species, RSV: resveratrol, SIRT1: silent information regulator 1, SOD: superoxide dismutase, SREBF-1c: sterol regulatory element binding factor-1c, SREBP: sterol regulatory element-binding protein, STD: standard diet, TG: triglyceride, UCP2: uncoupling Protein 2. ↑: increased, ↓: decreased. * The measured phosphorylation residue is not mentioned in the article.

## Abbreviations

ACC	acetyl-CoA-carboxylase
ACOX1	Acyl-Coenzyme A oxidase 1
AICAR	5-Aminoimidazole-4-carboxamide ribonucleotide
AKT	Protein kinase B
ALP	Alkaline phosphatase,
ALT	Alanine aminotransferase
AMP	Adenosine monophosphate
AMPK	5′AMP-activated protein kinase
AST	Aspartate aminotransferase
ATGL	Adipose triglyceride lipase
ATP	Adenosine triphosphate
BW	Body weight
CaMKKβ	Ca^2+^/calmodulin-dependent protein kinase kinase β
cAMP	Cyclic adenosine monophosphate
CHREBP	Carbohydrate response element binding protein
CPT	Carnitine palmitoyl transferase
CS	Citrate synthase
FAS	Fatty acid synthase
FATP5	Fatty acid transport protein 5
FFA	Free fatty acid
FOXO 1	Forkhead box protein O1
GGT	Gamma glutamil transpeptidase
GPAT1	Glycerol-3-phosphate acyltransferase 1
G6PDH	Glucose-6P-dehydrogenase
GSH	Glutathione
GPx	Glutathione peroxidase
HBV	Hepatitis B virus
HFD	High-fat diet
HFHS	High-fat high-sucrose diet
HSL	Hormone sensitive lipase
IkBα	Inhibitor of nuclear factor *kappa* subunit α
IL	Interleukin
LDH	Lactate dehydrogenase
LFD	Low fat diet
LKB1	Liver kinase B1
LXR	Liver X receptor
MCAD	Mitochondrial medium-chain acyl-CoA dehydrogenase
MDA	Malonaldehyde
ME	Malic enzyme
MTP	Microsomal triglyceride transfer protein
NAD^+^	Oxidized nicotinamide adenine dinucleotide
NADH	Reduced nicotinamide adenine dinucleotide
NAFLD	Non-alcoholic fatty liver disease
NF-kB p65	Nuclear factor kappa-light-chain-enhancer of activated B cells
O+A	Oleic acid *plus* alcohol
PDE	Phosphodiesterase
pIkBα	Phospho-inhibitory subunit of NF-KBα
PPAR	Peroxisome proliferator-activated receptor
PRKA	Protein kinase A
ROS	Reactive oxygen species
RSV	Resveratrol
si RNA	Small interfering RNA
SIRT1	Silent information regulator 1; Surtuin-1
SCD	Stearoyl-Coenzyme A desaturase 1
SQSTM1	Sequestosome 1
SOD	Superoxide dismutase
SREBF	Sterol regulatory element-binding transcription factor
SREBP	Sterol regulatory element-binding protein
STD	Standard diet
TG	Triglyceride
TNF-α	Tumor necrosis factor- α
UCP	Uncoupling protein
